# Trends in inequality of opportunity in health over the life cycle: The role of early-life conditions

**DOI:** 10.1016/j.jebo.2022.07.018

**Published:** 2022-09

**Authors:** Matija Kovacic, Cristina Elisa Orso

**Affiliations:** a European Commission, Joint Research Centre (JRC), Ispra, Italy; b Department of Law, Economics, and Cultures, Insubria University, Como, Italy

**Keywords:** Inequality of opportunity, Health, Life cycle, Adverse early-life conditions, Decomposition

## Abstract

This paper explores the evolution of inequality of opportunity in the prevalence of chronic diseases along the life cycle and across different birth cohorts for individuals aged 50 or older and residing in 13 European countries. We adopt an ex-ante parametric approach and rely on the dissimilarity index as our reference inequality metric. In addition to a commonly used set of circumstances, we pay particular attention to the role of adverse early-life conditions, such as the experience of harm and the quality of the relationship with parents. In order to quantify the relative importance of each circumstance, we apply the Shapley inequality decomposition method. Our results suggest that inequality of opportunity in health is not stable over the life cycle - it is generally lower at younger ages and then monotonically increases. Moreover, it varies between different birth cohorts and is generally higher for younger individuals than for older age groups. Finally, the contribution of adverse early life conditions ranges between 25% and 45%, which is comparable to the share of socio-economic circumstances but significantly higher than the relative contribution of other demographic characteristics, especially at younger ages.

## 1 Introduction

Following the call for health equity by the World Health Organisation (WHO, Commission on Social Determinants of Health 2008), the reduction of health inequalities represents a crucial goal for policy makers worldwide. In the European context, the WHO promoted the Health 2020 project with the aim of supporting action by government and society in order to: “significantly improve the health and well-being of populations, reduce health inequalities, strengthen public health and ensure people-centred health systems that are universal, equitable, sustainable and of high quality”.^1^

Health inequality may come from different sources, not all of which are equally objectionable. Based on the theoretical approach of Roemer (1998) to equality of opportunity, there is a distinction between “legitimate” and “illegitimate” sources of inequality. While legitimate sources of disparities can be attributed to consequences of individual effort (*i.e.,* determinants within individual control), illegitimate sources of differences are related to circumstances (*i.e.,* determinants beyond individuals’ responsibility) such as race, gender, ethnicity, characteristics of the neighborhood in which an individual grows, parental backgrounds and/or early-life conditions. Thus, inequality from given circumstances should be compensated while the one arising from efforts, which are mainly determined by individual choices and behavior, are morally and normatively accepted, and should be rewarded.

There are two approaches to the measurement of inequality of opportunity (IOp, henceforth): ex-ante and ex-post (Fleurbaey, Peragine, 2013, LiDonni, Peragine, Pignataro, 2014, Davillas, Jone, 2020). The ex-post approach seeks equality of outcomes between individuals who exert the same level of effort, irrespective of their circumstances (Roemer, 1998, Peragine, 2002, Aaberge, Mogstad, Peragine, 2011). The ex-ante approach, on the other hand, suggests that there is equality of opportunity if all individuals encounter the same set of opportunities, prior to their efforts and outcomes being realised (Ooghe, Schokkaert, Van der gaer, 2007, Bourguignon, Ferreira, Menedez, 2007, Checchi, Peragine, 2010, Fleurbaey, Peragine, 2013, Davillas, Jone, 2020). Empirically speaking, the ex-ante approach can be implemented using information on observed circumstances and outcomes since inequality is identified by comparing outcome distributions between different types defined in terms of circumstances. The total contribution of circumstances includes both the direct effect of circumstances on outcomes, and their indirect effect through efforts.

In this paper we adopt an ex-ante parametric approach and rely on a dissimilarity index to quantify the extent of inequality of opportunity in the prevalence of chronic diseases. Our contribution to the existing literature is twofold. First, we include, among the circumstances, a novel battery of variables (so-called *Adverse Childhood Circumstances* - ACE, henceforth) describing emotional and physical abuse in childhood, such as physical harm from parents and/or third parties, child neglect and the quality of the relationship with parents. We are particularly interested in disentangling the relative contribution of ACE to the overall IOp in the prevalence of chronic diseases both over the lifespan and in different birth cohorts. Individuals who experienced ACE are at increased odds of adverse health outcomes during life, such as premature death, diabetes, stroke, depression, fair/poor health, myocardial infarction, asthma, disability, severe obesity, mental distress, and sexually transmitted diseases (Sonu, Post, Feinglass, 2019, Chanlongbutra, Singh, Mueller, 2018, Campbell, Walker, Egede, 2016, Felitti, Anda, Nordenberg, Williamson, Spitz, Edwards, Koss, Marks, 1998.)

Second, we provide a comprehensive picture of the evolution of IOp in health during the lifespan for individuals born in different historical and economic contexts, and we discuss potential mechanisms linking early life conditions and health outcomes later in life. Unlike the existing literature which focuses mainly on a limited number of case-studies, we carry out a wide cross-country analysis including 13 European countries, namely Austria, Germany, Sweden, Spain, Italy, France, Denmark, Switzerland, Belgium, Czech Republic, Slovenia, Estonia, and Croatia. The sample consists of individuals born starting from the World War II (1940–45) and the immediate post-war period (1946–51) to more recent years (1952–57; 1958–63), in which most European countries have witnessed a significant increase in individual well-being and income. In order to explore the evolution of IOp over the life cycle, within each birth cohort, we quantify inequalities of opportunity arising at different life stages, namely when individuals were 25, 30, 35, 40, 45, 50, 55, 60 and 65 years old. In this way we are able to track changes in IOp starting from young adulthood to old age, separately for each birth cohort. For the purposes of our analysis, we use individual-level data drawn from the Survey of Health, Ageing and Retirement in Europe (SHARE). SHARE is a multidisciplinary, longitudinal survey on ageing which focuses on individuals aged 50+ and their spouses. Our analysis is based on retrospective information collected in the seventh wave of SHARELIFE, which allows us to track the health status of respondents over the life cycle, and to obtain information on an extensive set of childhood circumstances. With this information and coverage, the SHARE data set constitutes an ideal platform for the purposes of our analysis.

Looking at IOp over the life-cycle is crucial to understand how inequalities related to early- life conditions evolve over time. There is some evidence that risk factors associated with poor health and inappropriate living conditions tend to accumulate over a lifetime (Kim and Durden, 2007). This is in line with the so-called “cumulative advantage” hypothesis, suggesting that adverse circumstances and health disadvantages accumulate as individuals age. Conversely, other studies show that IOp in health increases with age up to a limit and then inequality begins to narrow most likely due to the “age-as-leveler” hypothesis (Davillas and Jone, 2020) which suggests that unavoidable biological processes may dominate the socio-economic determinants of health when older. Moreover, the evidence of generational differences in terms of IOp is important to properly monitor trends in health disparities among individuals exposed to different economic and social conditions, such as changes in the health and welfare systems in the countries where they live (van Kippersluis et al., 2009).

Overall, our findings support the “cumulative advantage” hypothesis since IOp is shown to increase monotonically over the life cycle. Moreover, we find a significant variation of IOp between birth cohorts. Indeed, with a few exceptions, inequality seems to be generally higher for younger than older cohorts. Regarding circumstances, there is a significant heterogeneity in terms of their contribution to inequality of opportunity in health: ACE account for a significant portion of inequality (from about 25% to almost 45%) and their contribution seems to be more pronounced at younger ages. Socio-economic conditions such as having experienced economic difficulties during childhood and parental education are also important and their contribution persists throughout life. Finally, the relative importance of demographic factors rises over the lifespan, especially for the oldest generation.

## 2 Related literature

A growing body of literature has addressed the measurement of IOp in health using different approaches and focusing on different countries (i.e., Davillas, Jone, 2020, Carrieri, Davillas, Jones, 2020, Rosa Dias, 2009; Fajardo-Gonzalez, 2016; Jusot et al., 2010, among others). Empirical research is mainly driven by data availability. Most studies of the adult population are based on data from single countries in Europe, especially from UK while studies analysing inequalities of opportunity among children are based on low or middle-income countries and focus on children less than 5 years old.

A key issue in measuring IOp in health is the choice of the health indicator. The existing evidence comes mainly from self-assessed health (SAH) as the main input for the measurement of health inequality (e.g., Kerkhofs, Lindeboom, 1995, Rosa Dias, 2009, Doiron, Fiebig, Johar, Suziedelyte, 2015; Fajardo-Gonzalez, 2016; Bricard et al., 2020).^2^ Other studies have used a more specific measure of health in adulthood, *i.e.*, the incidence of self-reported long-standing illness or disability at a specific age (Jones et al., 2012). Recently, some papers have measured inequality of opportunity in health using bio-markers instead of self-reported health indicators. Davillas and Jone (2020), for instance, use blood-based bio-markers considered relevant for specific chronic health conditions such as obesity, high blood-pressure and diabetes. In the same vein, Carrieri et al. (2020) use a composite biological measure to capture several health dimensions such as blood-pressure, inflammation, blood sugar level and cholesterol.

Regarding the choice of circumstances, most studies deem the socio-economic background an illegitimate source of inequality in health. Davillas and Jone (2020) include in circumstances the educational attainment of individuals and their parents, parental occupation, and childhood language. They find that these characteristics explain a non-trivial part of inequality, along with age and gender. Rosa Dias (2009) measures inequality of opportunity in health in the UK, adopting an ex-post approach. He considers a large set of circumstances such as parental socio-economic status, grandparents socio-economic status, educational attainment, lifestyles and health status of parents. Other studies focus on specific circumstances such as parental occupation. For instance, Bricard et al. (2020) stress the importance of the father’s occupation as a childhood circumstance. They use data from the 1958 National Child Development Study which records individual health status at different ages over the lifespan. At all ages, they find that individuals born to a “professional”, “senior manager or technician” father report a better health status and have a lower mortality rate than individuals born to manual workers and individuals without a father at birth. Both studies (i.e., Bricard, Jusot, Trannoy, Tubeauf, 2020, Rosa Dias, 2009) quantify IOp over the lifespan using UK data, and look at the health status of individuals at different ages. Conversely, Yan et al. (2020) quantify IOp in health in China focusing on the health status of elderly Chinese respondents. Childhood conditions include a large set of information on demographic factors, parent’s health and health behaviors, family socio-economic status, relationship with parents (if parents ever hit the respondent), and self-reported health when respondents were children. By using the Shapley value decomposition approach, they show that childhood circumstances may explain up to 23 percent of health inequality in old age among multiple health outcomes (cognitive health, mental health, physical health, etc.)

Only few studies measure inequality of opportunity in health using a cross-country perspective. Bricard et al. (2013) measure and compare inequality of opportunity in health in different European countries using data from the Retrospective Survey of SHARELIFE, which focuses on life histories of European people aged 50 and over in 2008/2009. In particular, the paper investigates whether the correlation between effort (lifestyles) and circumstances (social conditions in childhood, parents’ longevity and parents’ health-related behaviors) differ from one country to another. Their findings suggest that inequalities of opportunity in health are mainly driven by social background affecting adult health directly, and so would require policies compensating for poorer initial conditions. In another interesting study, Jusot et al. (2010) use data from the same survey (SHARELIFE) to quantify inequality of opportunity in health in a set of European countries. They focus on health status in adulthood (self-assessed health), and show the existence of inequalities of opportunity in health among different European countries related to circumstances. In particular, inequalities of opportunity in health are particularly marked in Mediterranean and Germanic countries while in the Nordic countries they appear as less pronounced. Both studies assess inequality of opportunity in health focusing on the health status of respondents in adulthood (aged 50 or older).

## 3 Data and methodology

Individual-level data employed in this study are drawn from the Survey of Health, Ageing and Retirement in Europe (SHARE). SHARE is a multidisciplinary longitudinal survey on ageing which focuses on individuals aged 50+ and their spouses. The survey contains both regular and retrospective waves (SHARELIFE). The regular rounds collect information on the individuals’ current situation, such as health, working situation, social network/relations, accommodation, economic situation/assets, behavioral risks, and expectations. In addition, two survey rounds add retrospective information on multiple dimensions of the respondents’ past (health, health care, accommodation, working career, household situation and performance at school during childhood, number of children, childbearing for women, emotional experiences in early life, relationship with parents, adverse childhood experiences, etc.).

What makes SHARE data particularly suited for the purposes of our analysis is the ability to link the information on the respondents’ current situation to retrospective childhood/adulthood data. First, using retrospective data enables us to track the health status of respondents over the lifespan. We create a set of variables describing the number of chronic conditions an individual has reported suffering from at different ages (*i.e.*, 25, 30, 35, 40, 45, 50, 55, 60, 65). The chronic conditions considered are the following: heart diseases (including angina, heart attack or other heart problems), stroke, diabetes or high blood sugar, lung disease other than asthma (e.g. bronchitis, chronic obstructive pulmonary disease or tuberculosis), cancer or malignant tumour, Parkinson’s disease, Alzheimer’s disease, emotional distress, kidney disease, high blood pressure, high cholesterol, asthma, arthritis (including osteoarthritis and rheumatism), osteoporosis, ulcer, nervous or psychiatric problems, and cardio-vascular diseases. We then generate a dummy indicator assigning value 1 whether an individual reports suffering from at least one chronic condition listed above. In addition, we also compare the number of chronic diseases with the median prevalence of comorbidities at the country-cohort level and for each life-stage by means of a binary variable with value 1 if the number of diseases declared by individuals is higher than or equal to the median of their generation at each specific age, and zero otherwise.^3^

Second, retrospective data allows us to consider an extensive set of childhood circumstances. We are particularly interested in disentangling inequalities by means of a specific set of early-life conditions called “*Adverse Childhood Circumstances*”. The retrospective SHARELIFE component of the survey asks respondents to report information on exposure to child neglect and childhood physical abuse, either from mother, father or third parties. More precisely, the questionnaire asks the following questions:

1. How often did your mother/your father push, grab, shove, throw something at you, slap or hit you? 1. Often 2. Sometimes 3. Rarely 4. Never.
2. How often did anybody else physically harm you in any way? 1. Often 2. Sometimes 3. Rarely 4. Never.;
3. How much did your mother/your father (or the woman/man that raised you) understand your problems and worries? 1. A lot 2. Some 3. A little 4. Not at all.
4. How would you rate the relationship with your mother/your father (or the woman/man that raised you)? 1. Excellent 2. Very good 3. Good 4. Fair 5. Poor.

We consider that an individual experienced physical abuse from either the mother or the father during childhood if s/he answers “1. Often” or “2. Sometimes” to question 1. We treat question 2 in the same manner to capture physical harm from other persons. A situation of “child neglect” corresponds to answers “3. A little” or “4. Not at all” to question 3. The relationship with mother/father in childhood is rated 1, i.e.,problematic/negative, if the respondent answers “4. Fair” or “5. Poor” to the last question. Both “child neglect” and relationship variables describe the quality of parent-child relationships. Since they are highly correlated, we opt for including only one, namely the rating of the relationship with parents.^4^

Among the circumstances, we also include gender, absence of a parent, financial hardship during childhood, parental education, household size, and health status when the respondent was ten years old. Concerning childhood health, the following self-assessed health (SAH, henceforth) status question was asked: “Would you say that your health during your childhood was in general excellent, very good, good, fair, or poor?”. SAH was therefore measured on a five-point scale from “excellent” (score 5) to “poor” (score 1) and treated as an ordered categorical variable. It was dichotomized into a binary variable with value 1 if individuals declare that their health during childhood was fair or poor, and 0 otherwise. As for the parental financial condition, the respondents were asked whether their family was fairly well off financially, about average or poor. We use a binary variable with a value of 1 assigned to individuals reporting early-life financial hardship. Finally, the highest level of parental education establishes whether one or both parents hold a tertiary degree (as defined by ISCED-97). Table A.2 (in Appendix) reports summary statistics for the set of circumstances and chronic conditions variables.

### 3.1 Sample and cohorts

Regarding the analysis of IOp over the generations, we consider four contiguous 6-year cohorts of individuals born from World War II (1940–45) and the immediate post-war period (1946–51) to more recent years (1952–57; 1958–63). As a robustness check we also consider alternative specifications of cohorts defined at a 5-year (1941–45; 1946–50; 1951–55; 1956–60), 6-year (1939–44; 1945–50; 1951–56; 1957–62) and 10-year intervals (1935–44; 1945–54; 1955–1964). Even though the year of birth of the respondents in SHARE spans from the 1920s to 1970s, the choice of cohort partitions was mainly driven by two concerns, namely the requirement of a balanced distribution of individuals between cohorts and overall country coverage. The trade-off between these two objectives was significantly influenced by data availability.

In order to increase the total number of observations and to guarantee a meaningful comparison of IOp between generations and during the life cycle, we consider a panel of individuals interviewed starting from wave 4 and also present in wave 7. For eight countries that joined SHARE only in 2017 (Lithuania, Bulgaria, Cyprus, Finland, Latvia, Malta, Romania and Slovakia) together with Hungary (which participated in waves 1 and 7) and the Netherlands (not present in wave 7), we do not have information on parental backgrounds (collected in waves 5 and 6 but not in wave 7), and a further three countries were excluded due to insufficient data coverage (Greece, Poland and Portugal), while Israel was not considered because of the particularities of the sample composition (more than 55% of foreign-born respondents). In order to produce meaningful and comparable evidence on the evolution of IOp over time, we also had to assure a satisfactory balance between all the birth cohorts considered in terms of data coverage and composition. In some cases (Luxembourg) this was not possible. As a consequence, Luxembourg was excluded from the analysis. Our final sample covers 13 European countries for a total of 92,960 individuals (6-year cohorts) for which the information on the prevalence of chronic diseases was collected in 2017 (wave 7).^5^ Table 1 shows the distribution of individuals across countries and cohorts (6-year partition) in the sample used as a baseline specification.^6^

**Table 1:** Number of observations by country and cohort.

Country	1940–45	1946–51	1952–57	1958–63
Austria	2356	2475	2230	1496
Germany	1612	2270	2277	2719
Sweden	1794	1879	1486	720
Spain	1957	2245	2426	1895
Italy	1493	1890	2088	2196
France	1133	1825	2012	1674
Denmark	828	1165	1477	2307
Switzerland	1342	1468	1819	1164
Belgium	1359	2091	2771	3020
Czech Republic	2765	3556	2947	1293
Slovenia	2225	2934	3162	1708
Estonia	3920	4317	4096	2661
Croatia	862	1193	1338	1224

*Notes: Authors’ processing of SHARE data, waves 4–7*.

Birth cohorts considered in the analysis were exposed to different socio-economic and historical conditions. Unlike for younger cohorts (born after 1950), the older generations (late 1930s and 1940s) include individuals born immediately before, during or after World War II. Experience with the war and the financial hardship immediately afterwards may have had important effects on outcomes later in life (Kesternich et al., 2014). First, the war caused severe hunger crises which led to many casualties, and may have had long-term effects on the health status of survivors. Second, older cohorts may have been exposed to negative events such as dispossession, persecution and migration related to the war. Dispossession was often associated with persecution and resulted in the geographic displacement of populations. All these exogenous circumstances led to severe economic and emotional insecurity, which in turn could have prevented individuals from achieving good health during their lives.

### 3.2 Empirical strategy and inequality metric

In order to quantify the extent of inequality of opportunity across the life cycle and between cohorts, we estimate a set of country-cohort level regressions for each specific age at which the prevalence of chronic diseases is recorded. Thus, we obtain a point estimate of inequality of opportunity at different stages of the life cycle, separately for each birth cohort. More precisely, we estimate the following reduced-form logit model:

(1) $$P\left(y_{i}\right)=\frac{\exp(r_{i})}{1+\exp(r_{i})}$$

where:

$$r_{i}=\alpha +\beta {\mathrm{CI}}_{i}+\epsilon_{i}.$$

where $y_{i}$ is a dummy variable that equals 1 if an individual i reports suffering from at least one chronic disease, and 0 otherwise, and CI is a vector of circumstances. Coefficients β reflect the total contribution of circumstances and include both the direct effect of circumstances on outcomes, and their indirect effect through efforts. We then generate a counterfactual distribution {y} where y is replaced with its predicted value $\hat{y}$. Since the predicted outcomes are the same for all individuals with identical circumstances (Ferreira and Gignoux, 2011), we can estimate the absolute IOp by means of an inequality metric applied to the distribution of the predicted values, $\hat{D}$.^7^

As a reference inequality metric, we calculate a dissimilarity index. This index can be defined as a measure proportional to the absolute distance between the distribution of circumstances among those with high outcomes (*i.e.*, not suffering from any chronic disease) and the distribution among those with low outcomes (suffering from at least one chronic disease). Following Paes de Barros et al. (2008) and Fajardo-Gonzalez (2019), a consistent estimator for the dissimilarity index for dichotomous outcomes is given by:

(2) $$\hat{D}=\frac{1}{2\bar{y}}\sum_{i=1}^{n}l_{i}\left|\hat{y_{i}}-\bar{y}\right|$$

where $\hat{y_{i}}$ is the predicted probability of suffering from one or more chronic disease for individuals of type (circumstance group) i=1,…,n, while $\bar{y}=\sum_{i=1}^{n}l_{i}\hat{y_{i}}$ stands for the estimated conditional probability with $l_{i}=1/n$ denoting sampling weights.

The index $\hat{D}$ can be interpreted as the minimum fraction of healthy individuals (*i.e.*, those with no chronic diseases) that needs to be redistributed across circumstance groups in order to achieve equal opportunity (*i.e.*, when an equal proportion of people with some chronic conditions are found in all circumstance groups). The index ranges from 0 to 1 and takes the value zero when opportunities are spread evenly throughout the population (Fajardo-Gonzalez, 2019). Since we are not able to account for all potential determinants beyond individuals’ responsibility, the index provides a lower-bound estimate of the overall inequality due to all circumstances, not only those that are observed (Davillas and Jone, 2020; Ferreira and Gignoux, 2011).

### 3.3 Decomposition of the dissimilarity index

The ex-ante parametric approach presented so far provided us with a point estimate of absolute inequality of opportunity in the prevalence of chronic diseases at different stages of the life cycle. In order to better understand the phenomenon of interest and its evolution over time, we decompose inequality of opportunity in a given country and for different cohorts by estimating the relative importance of each circumstance using the Shapley value. Hence, we are able to divide inequality of opportunity into its components and attribute a part of total IOp to each circumstance. While interpreting the results, it is important to bear in mind that the results obtained *cannot* be interpreted as causal but they represent a proxy of the relative importance of each circumstance. As suggested by Ferreira and Gignoux (2014), the process of decomposition may suffer from multicollinearity since most of the circumstances are often correlated. It is important to note, however, that the presence of multicollinearity does not influence the precision of the calculated inequality of opportunity measures.

The relative contribution of a circumstance is given by the average change in inequality of opportunity when this circumstance is added to the model over all possible inclusion sequences. If we denote with CI the entire set of N circumstances arranged in some order CI∈{1,…,c,…N}, and with S⊂CI any randomly selected subset of M circumstances, then the marginal contribution of any circumstance c∈S to the value of the dissimilarity index $\hat{D}\left(S\setminus \left\{c\right\}\right)$ is defined by $\hat{D}\left(S\right)-\hat{D}\left(S\setminus \left\{c\right\}\right)$. The probability distribution over S is given by the product of the probability that a circumstance c is in the Sth place (which is simply equal to 1/N) and the probability that S∖{c} actually occurs when we randomly select M−1 circumstances from the population CI∖{c}. This probability is simply given by (N−M)!(M−1)!/(N−1)!. The relative contribution of any circumstance c∈CI to the value of $\hat{D}$ is given by:

(3) $$RC_{c}\left(CI,\hat{D}\right)=\frac{\sum_{S\subset CI,c\in S}\frac{(N-M)!(M-1)!}{(N-1)!}\left[\hat{D}\left(S\right)-\hat{D}\left(S\setminus \left\{c\right\}\right)\right]}{\hat{D}\left(CI\right)}$$

with $\sum RC_{c}\left(\cdot \right)=1,c=1,\ldots ,N$.

## 4 Results

### 4.1 Evolution of inequality of opportunity in health over time and throughout the life cycle

**Fig. 1:**
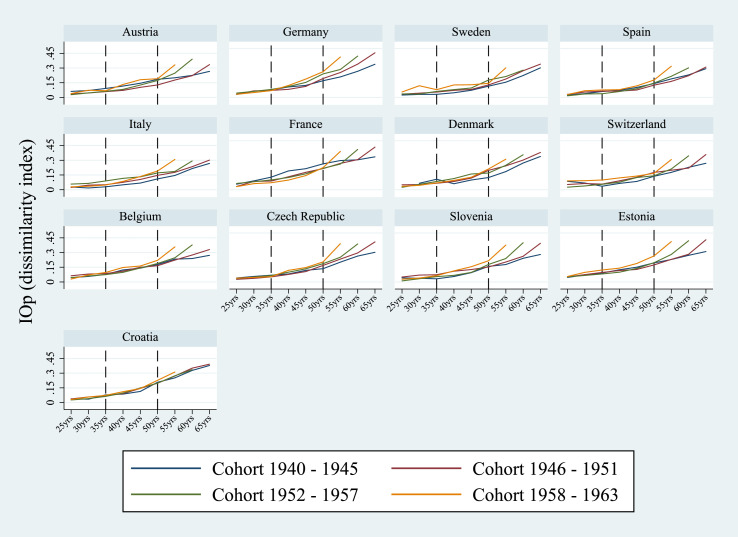
IOp in the prevalence of chronic diseases over the life cycle, by country and cohort. *Notes:* Authors’ processing of SHARE data, waves 4–7. The value for IOp for Denmark for the oldest cohort (1940–45) at the age of 25 was excluded due to the insufficient prevalence of chronic diseases in the population.

Fig. 1 and Table A.1 (in the Appendix) show IOp in health over the lifespan for each of the four cohorts considered. The levels of absolute inequality of opportunity are calculated at different ages over the lifespan, namely when the individuals were 25, 30, 35, 40, 45, 50, 55, 60 and 65 years old. For the age of 65, the point estimates are shown only for the two oldest cohorts since the sub-sample of individuals born after 1952 and aged 65 at the time of the interview was too small to produce reliable estimates.

Regarding the evolution of IOp over the life cycle, in most countries we observe a gradual rise in inequality of opportunity with increasing age. One explanation for such a pattern may lie in the so-called “cumulative advantage” hypothesis according to which adverse circumstances and health disadvantages accumulate as individuals age (Davillas, Jone, 2020, Kim, Durden, 2007). This finding suggests a more significant role of circumstances in health as people age: small differences early in life can widen in the course of a lifetime, leading to accumulating health disadvantages later in life.^8^

Interestingly, inequality of opportunity tends to be more pronounced for younger cohorts than for older age groups, in particular for a subset of countries like Italy, Sweden, Germany, Spain, Czech Republic, Estonia and Slovenia. Generally, the divergence of IOp between cohorts is more pronounced after the age of 50 in almost all countries considered.

There are two possible explanations for this pattern. The first is related to the distribution of parental socio-economic conditions during the respondents’ childhood. Indeed, during the period of the Second World War and in the years immediately following the war, large portions of the population experienced similar circumstances in terms of financial hardship and precarious economic conditions. This regularity resulted in lower overall inequality, both in absolute terms and regarding opportunities. In such a context, countries with initially higher levels of development were also relatively more unequal in terms of outcomes and opportunities.

During the *Golden Age of Capitalism* from 1950 to 1969, however, the social and economic situation started to evolve and several European countries experienced a period of economic expansion at different speeds. Southern European countries like Italy and Spain, along with Germany, registered the fastest growth rates worldwide (between 5.5% and 6.5%).^9^ The widely documented evidence underlying the inverted “U” shape relationship between economic development and inequality (Kuznets curve) resulted in more asymmetric exposure to adverse conditions between social groups, making some individuals initially more disadvantaged than others. Since parental socio-economic conditions are inherited and are beyond an individual’s control, higher overall inequality may have been accompanied by a greater disparity in opportunities.

**Fig. 2:**
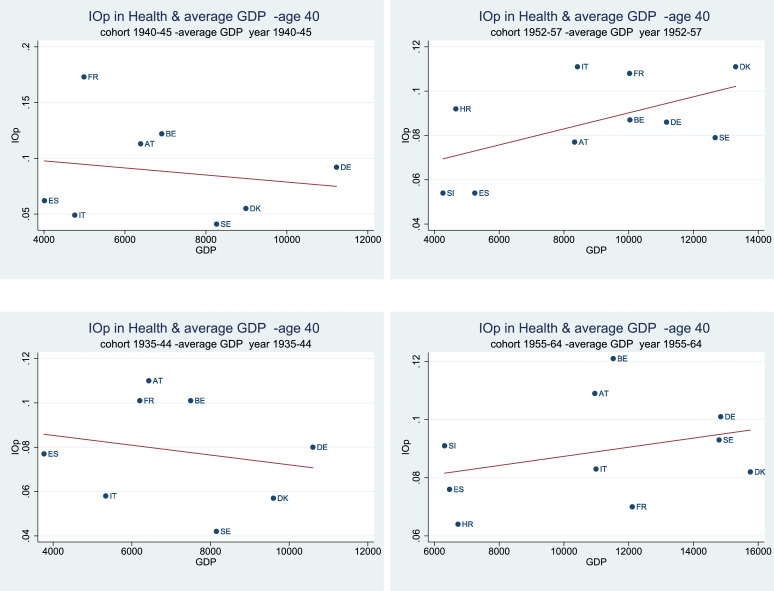
IOp in the prevalence of chronic diseases over the life cycle, and average GDP at birth - age 40. *Notes:* Authors’ processing of SHARE data, waves 4–7, and Brugiavini and Weber (2014). The estimated correlations are statistically significant at the 1% level for younger generations (1952–57; 1955–64), while they are significant at 10% for individuals born in the cohort 1935–44. Regarding individuals born between 1940 and 1945, correlations are not statistically different from zero.

**Fig. 3:**
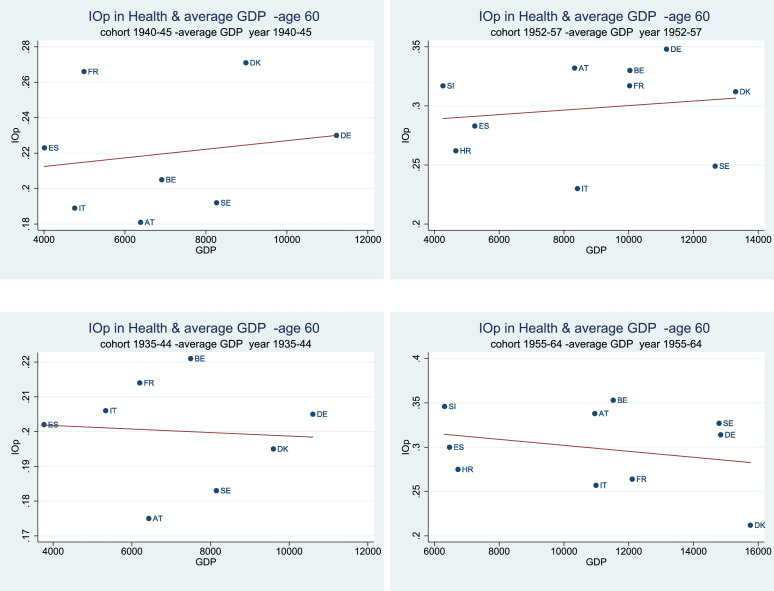
IOp in the prevalence of chronic diseases over the life cycle, and average GDP at birth - age 60. *Notes:* Authors’ processing of SHARE data, waves 4–7, and Brugiavini and Weber (2014). The estimated correlations are not statistically different from zero in all cases, with the exception of the cohort 1955–64.

In support of this conjecture, Figs. 2 and 3 show the correlation between IOp in health for two different birth cohorts, and the average GDP measured over the same period. The choice of the cohorts (both at a 6-year and a 10-year interval) overlaps with the periods prior to (1940–45; 1935–44) and during the post-war economic expansion (1958–63; 1955–64). There is a positive relationship between IOp and GDP starting from the beginning of the *Golden Age of Capitalism* early in life (age 40) while the relationship tends to become less clear (flat or slightly negative) at the age of 60. The fact that the link between opportunities and development weakens with age is not surprising, since at later life stages the individuals are more affected by other factors, such as experience, life-style, improved medical care and their overall economic well-being which is assumed to increase with age.

Another potential explanation for higher levels of inequality among younger generations may be related to mortality, which might reduce the magnitude of IOp across different birth cohorts, emphasizing the gap between old and young generations. Indeed, individuals experiencing poorer initial conditions may die prematurely, and there may be a selection bias since the oldest cohorts in the sample include individuals with better health prospects and, hence, with longer life expectancy (Bricard et al., 2020).

**Fig. 4:**
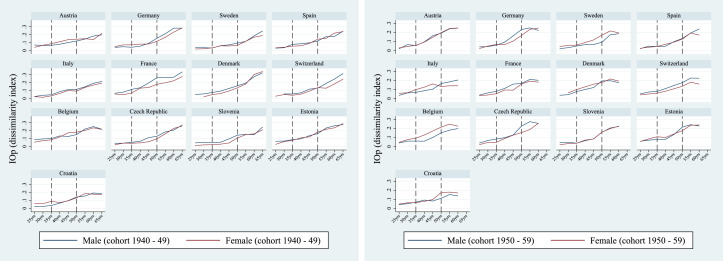
IOp in the prevalence of chronic diseases over the life cycle, by cohort and gender. *Notes:* Authors’ processing of SHARE data, waves 4–7.

An interesting picture emerges when we look at differences in IOp between males and females. Fig. 4 shows the evolution of IOp over the life cycle separately for men and women born in two different historical contexts, namely during and immediately after World War II (cohort 1), and in the 50s (cohort 2). We were not able to carry out the analysis by gender for four different cohorts as in Fig. 1 because the sample coverage was not sufficient to produce reliable estimates of inequality of opportunity separately for males and females. There is an evident gender gap in most countries: among individuals belonging to older age groups, IOp is generally higher for men than for women, while for younger cohorts we observe reversal of the trend with women experiencing more unfairness than men. Once again, this discrepancy may be due to social and economic development during the post-war period, in particular in terms of women’s empowerment. Overall, women born after 1950 experienced different socio-economic conditions than women born earlier. In particular, the societal changes in the orientation towards paid employment driven by increased education or by a drop in fertility may have translated into higher rates of labor force participation experienced by younger cohorts of women (Euwals et al., 2011). As a consequence, improvements in the socio-economic status of specific sub-groups of women may have exacerbated inequalities in health between different generations. This gender-based switch in IOp is particularly pronounced in Sweden, Denmark, Belgium, and Italy (up to 45). This may be surprising in part, especially for Nordic countries, which have shown high levels of gender equality in recent decades. A possible (speculative) explanation for that may lie in the process of women’s empowerment, which was particularly pronounced in these countries during the post-war period. Statistics on female employment rates, for instance, document substantial improvements in countries like Sweden and Denmark from the 60s to the 80s.^10^ This process, especially at the initial stage, might have favoured specific sub-groups of women (for instance, those who experienced better childhood conditions) and consequently exacerbated inequalities of opportunity in health between different generations.

### 4.2 Decomposition of IOp: The role of ACE

**Fig. 5:**
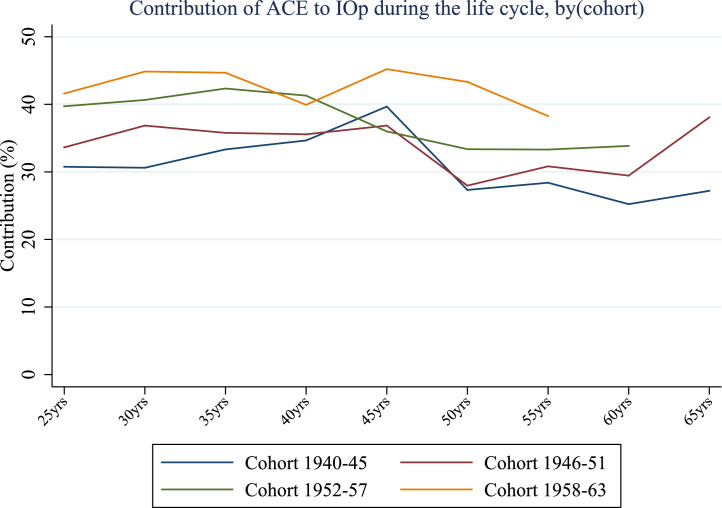
Relative contribution of ACE (%) over the life cycle, by cohort (cross-country average). *Notes:* Authors’ processing of SHARE data, waves 4–7.

Together with the evolution of inequality of opportunity over time documented in the previous section, one of the main objectives of this research is to quantify the relative contribution of parent-child relationship variables, and other demographic and socio-economic circumstances, to disparities in the prevalence of chronic diseases.^11^ Fig. 5 shows the average contribution of ACE at each stage of the life cycle, by cohort. This novel set of circumstances represents an important source of inequality of opportunity over the lifespan for all cohorts considered. Indeed, the relative contribution of ACE ranges (roughly) between 25% and 45% on average, with a gradually decreasing trend over the life cycle.

Interestingly, ACE appear relatively more important for younger cohorts (1952–57; 1958–63), and especially in young adulthood. The contribution of ACE for the youngest generation is on average 10 percentage points higher than for the oldest cohort. From the age of 45 IOp follows a decreasing trend while remaining approximately at the same distance with respect to older generations. One possible explanation may be related to differences in reporting physical and emotional abuse across cohorts. It may be that changes in norms and attitudes over time about how parents bring up their children mean that what was considered simply strict parenting in one era is deemed abuse in another. For example, commonly held views of what constitutes physical abuse during the war might have changed in the post-war period (Attanasio et al., 2020).

**Fig. 6:**
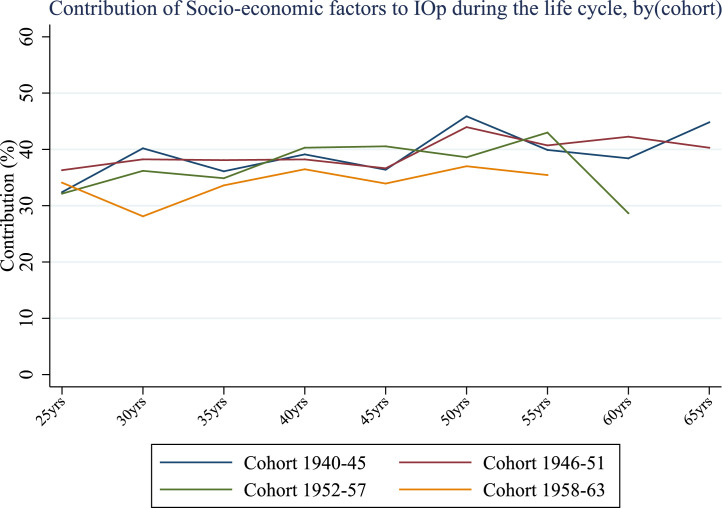
Relative contribution of socio-economic conditions (%) over the life cycle, by cohort (cross-country average). *Notes:* Authors’ processing of SHARE data, waves 4–7.

**Fig. 7:**
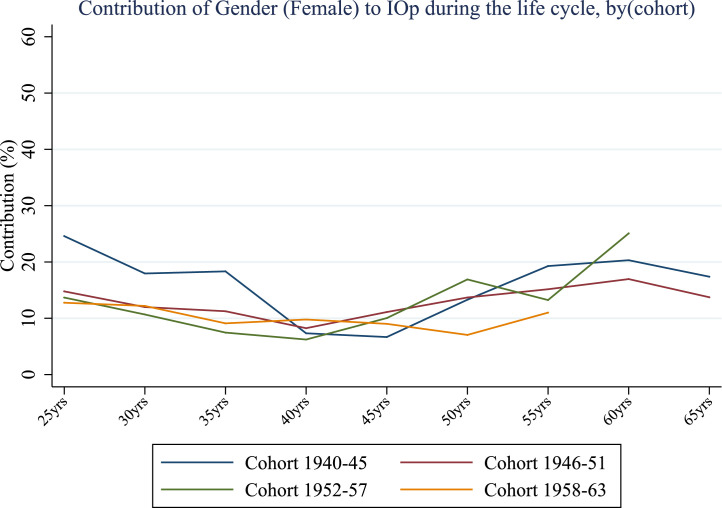
Relative contribution of gender (%) over the life cycle, by cohort (cross-country average). *Notes:* Authors’ processing of SHARE data, waves 4–7.

It is also worth noting that the relative importance of ACE decreases over the life cycle for all generations except for the immediate post-war cohort. Compared to the evidence emerging from Fig. 1, a more pronounced increase in inequality of opportunity after the age of 45 is not followed by an equivalent increase in the contribution of ACE; instead, older life stages see socio-economic factors gaining more importance at the expenses of ACE (Fig. 6). This is not surprising since the effects of early life conditions may fade later in life while other health-related factors, presumably more persistent and dependent on parental socio-economic backgrounds,^12^ may win the race in determining the magnitude of IOp. When individuals are younger, the influence of childhood conditions is stronger, not necessarily because of the shorter time between their occurrence and chronic disease but because some other circumstances may gain relevance in middle or old-age. Indeed, the evidence in Figs. 6 and 7 confirms the interplay between ACE and other circumstances - the relative importance of gender gradually decreases up to the age of 40 for all birth cohorts and then rises by 10 to 15 percentage points at the age of 60. This is a very interesting result that highlights significant unfairness in the distribution of opportunities of good health outcomes for women.

Overall, the fraction of inequality that can be attributed to ACE is significant. The impact of early life circumstances related to the parent-child relationship is comparable to the share of socio-economic circumstances such as financial hardship, parental education, household size and the absence of a parent, while it significantly exceeds the impact of gender, especially in young adulthood.

**Fig. A.2:**
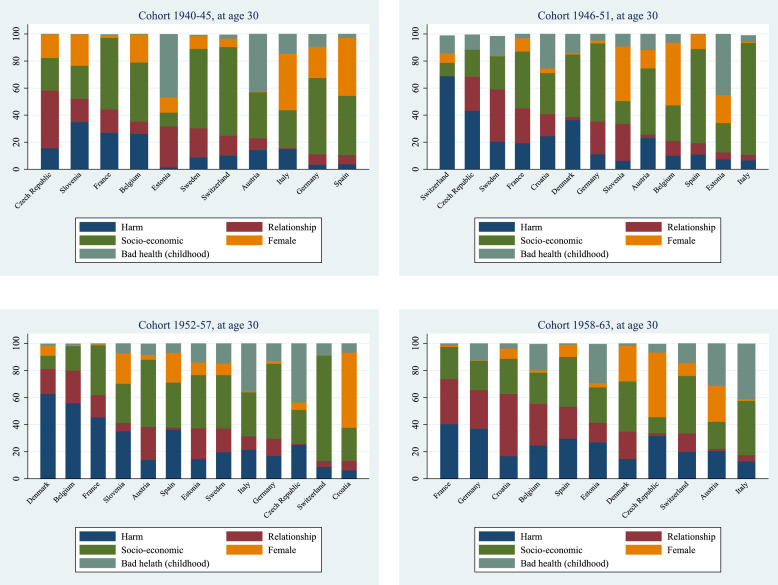
Relative contribution of circumstances (%) at age 30, by country and cohort. *Notes:* Authors’ processing of SHARE data, waves 4–7. Contributions for Denmark and Croatia for the youngest and oldest cohorts (1940–45; 1958–63) were excluded due to an insufficient number of observations (less than 500).

**Fig. A.3:**
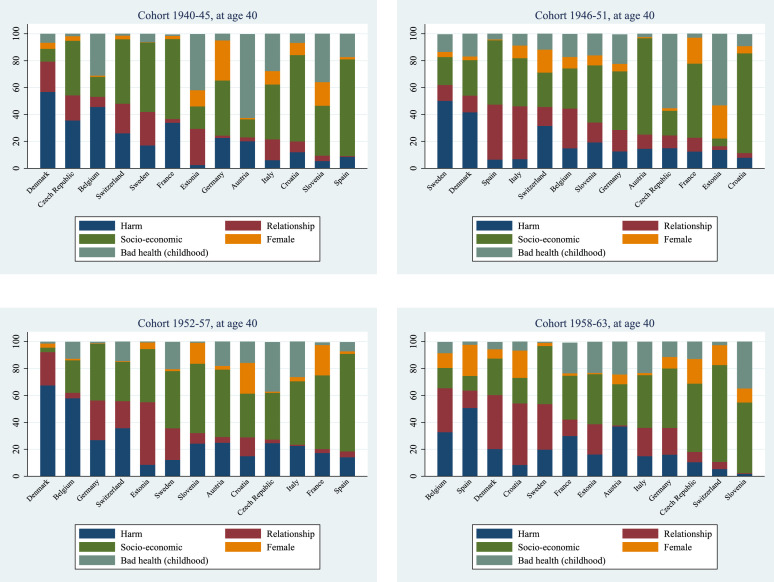
Relative contribution of circumstances (%) at age 40, by country and cohort. *Notes:* Authors’ processing of SHARE data, waves 4–7.

**Fig. A.4:**
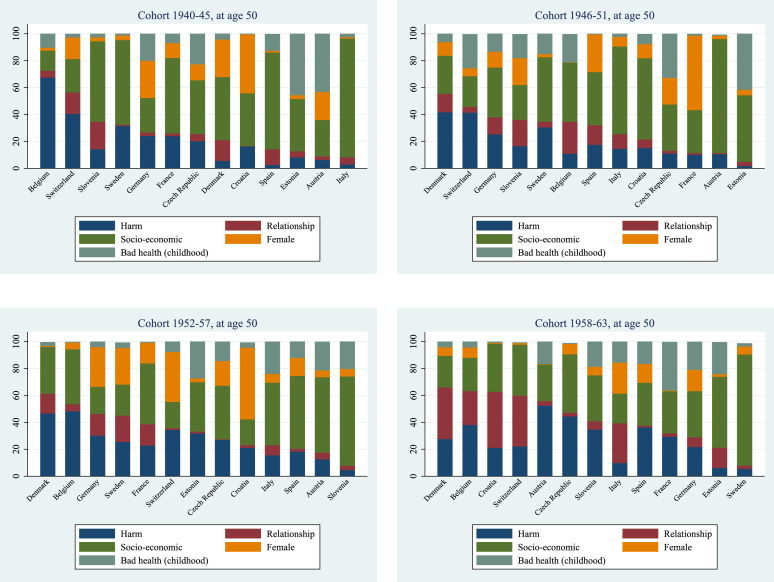
Relative contribution of circumstances (%) at age 50, by country and cohort. *Notes:* Authors’ processing of SHARE data, waves 4–7.

**Fig. A.5:**
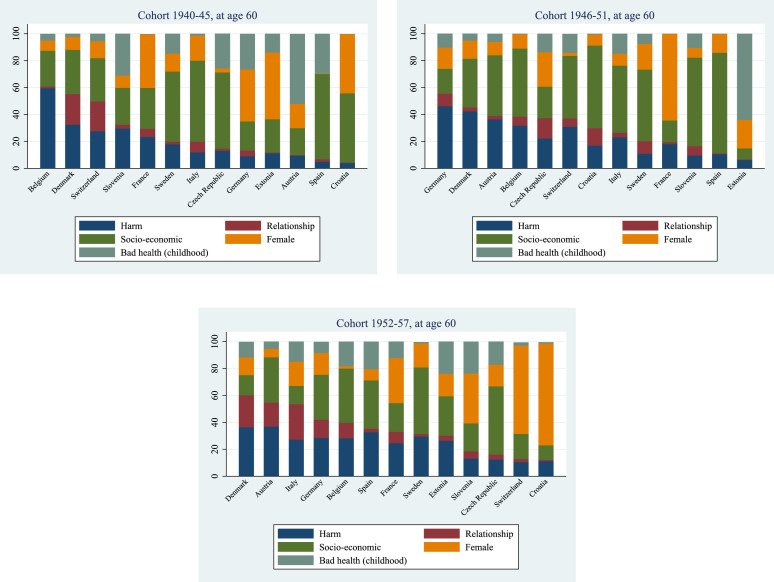
Relative contribution of circumstances (%) at age 60, by country and cohort. *Notes:* Authors’ processing of SHARE data, waves 4–7.

In spite of the relatively clear patterns of contributions across the lifespan and cohorts, significant heterogeneity exists between countries. Fig. A.2, Fig. A.3, Fig. A.4, Fig. A.5 (in Appendix) provide a more detailed insight into the relative importance of ACE along with the other circumstances, separately for each country and for each birth cohort. For the sake of space and clarity, we focus on four instead of nine distinct ages, namely 30, 40, 50, and 60. For the age of 60, we were able to measure contributions only for three consecutive cohorts (*i.e.*, 1940–45, 1946–51 and 1952–57). To offer a more precise picture of the relative importance of each ACE component, we split the category into two parts, namely “harm” (including both the harm from parents and third parties) and “relationship”. On the other side, we continue to consider socio-economic circumstances as an aggregate category. By comparing the contributions at each life-stage between different birth cohorts enables us to retrieve useful information on the evolution of the relative importance of each circumstance (or groups of circumstances) over the generations at different ages.

Overall, southern and south-eastern European countries such as Italy, Spain, and Croatia feature lower contributions of ACE while the relative importance of socio-economic conditions remains high, both in young adulthood (age 40) and later in life (age 50 and age 60). This evidence confirms the important role played by economic and social conditions during childhood in shaping individual opportunities for health later in life. The largest contributions of ACE are found in northern and some central European countries. The gap between countries at the upper and the lower bound is particularly pronounced for younger cohorts. Interestingly, cohorts where ACE is less important are offset by a higher incidence of socio-economic conditions and less by gender and adverse health conditions in early life.

Compared to adverse early life conditions, poor health conditions in childhood account for a smaller portion of IOp, especially in older cohorts (1940–45; 1946–51) and at later stages in life (at the age of 60), and the relative contribution is also rather negligible for younger individuals. An interesting finding emerges comparing the relative importance of ACE, socio-economic conditions in early life and bad health conditions in childhood: countries with a higher contribution of ACE are also those where bad health is relatively less important compared to other countries characterized by lower contributions of ACE.

Finally, the gender component accounts for a larger portion of inequality of opportunity at older ages (*i.e.*, when individuals are aged 60). This evidence seems to hold for all cohorts examined. As in the case of socio-economic conditions and bad health, being female is more important for IOp in countries where the contribution of ACE is relatively lower.

### 4.3 Potential pathways linking ACE and chronic diseases

As for the potential pathways by which ACE could influence health over the lifespan, behavioral factors may represent an important channel. A large body of literature has shown significant associations between exposure to ACE and smoking behavior in adulthood (Brugiavini, Buia, Kovacic, Orso, 2022, Buia, Kovacic, Orso, Borsch-Supan, Andersen-Ranberg, J. Bristle, Brugiavini, Jusot, Weber, 2019, Ford, Anda, Edwards, Perry, Zhao, Li, Croft, 2011; Case et al., 2005; Anda, Whitfield, Felitti, Chapman, Edwards, Dube, Williamson, 2002, Anda, Croft, Felitti, et al., 1999). One possible explanation for this association may be that nicotine has psychoactive benefits that could unconsciously help regulate stress in individuals exposed to ACE (Su et al., 2015). Another potential behavioral consequence of ACE is obesity (Danese, McEwen, 2012, Midei, Matthews, 2011, Greenfield, Marks, 2009). Potential mechanisms linking ACE with obesity include negative affect (anger, perceived stress) and disordered eating (Rohde, Ichikawa, Simon, Ludman, Linde, Jeffery, Operskalski, 2008, Greenfield, Marks, 2009). This evidence, together with the extensive literature showing a robust relationship between behavioural risks and chronic diseases (Lahiri et al., 2000; Larsson et al., 2002; Brick, 2004; Dixon, Johnston, 2010, Elwood, Galante, Pickering, Palmer, Bayer, Ben-Shlomo, Longley, Gallacher, 2013), may suggest that part of the effect of ACE is indirect and passes through individual unhealthy behaviour. However, further research is needed to explore these mechanisms in more detail.

Other possible pathways linking ACE to health over the life cycle may involve biological factors. Medical studies have shown how childhood adversities, such as physical and emotional abuse, may cause alterations in multiple systems that regulate stress responses, including nervous, neuro-endocrine, and immune systems (Su, Jimenez, Roberts, Loucks, 2015, Nusslock, Miller, 2016). It is known that the human brain is not fully developed at birth and undergoes important changes through to young adulthood (Buss et al., 2012). Childhood trauma can negatively affect the structure and function of the brain. At the same time, some studies have shown that alterations in the nervous, neuro-endocrine, and immune systems may impact on the mental and cardiovascular health of individuals (Nusslock, Miller, 2016, Danese, McEwen, 2012).

Along with non-biological factors such as childhood circumstances, other biological characteristics such as genetic predisposition importantly contribute to the insurgency of specific diseases. Existing evidence supports the role of both biologic and non-biologic contributors in the development of chronic diseases from early life (Panasevich, Melén, Hallberg, Bergström, Svartengren, Pershagen, Nyberg, 2013, Stephens, Kessler, Buss, Miller, Grobman, Keenan-Devlin, Borders, Adam, 2021). It is well documented that some individuals are more vulnerable to specific chronic diseases and their risk factors. Such differences begin in early life. For instance, in recent years, different studies, including genome-wide association studies and meta-analyses, have identified common genetic variants that contribute to cardiovascular diseases (Kesternich et al., 2014). However, disentangling the contribution of genetic and environmental factors remains a challenging task.

### 4.4 Sensitivity analysis

The validity of our results is tested via a set of robustness checks. We concentrate on the measurement of absolute IOp in health across the lifespan, by country and cohort while, for the sake of space, we do not report the decomposition results, which are available from the authors upon request.

**Fig. 8:**
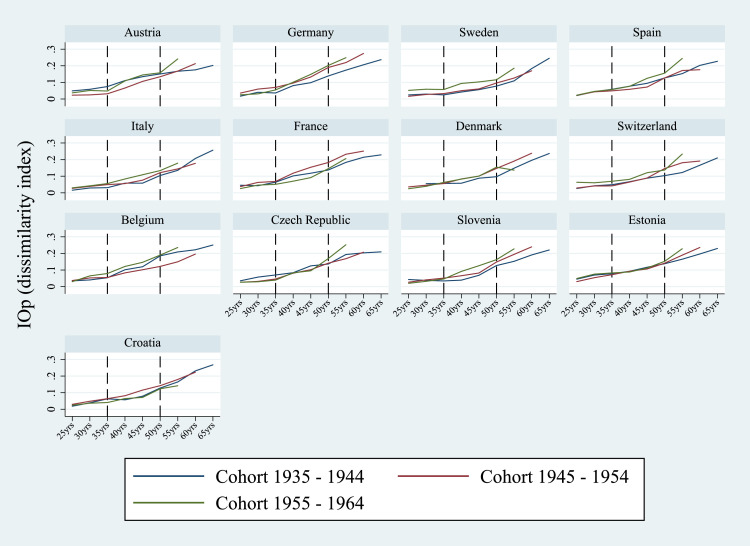
IOp in the prevalence of chronic diseases over the life cycle, 10-year cohorts. *Notes:* Authors’ processing of SHARE data, waves 4–7.

First, in order to test whether our results are sensitive to cohort partitions, we re-run the model by considering alternative cohort specifications, *i.e.*, four cohorts at a 5-year interval (1941–45; 1946–50; 1951–55; 1956–60), four cohorts at a 6-year interval (1939–44; 1945–50; 1951–56; 1957–62) and three cohorts at a 10-year interval (1935–44; 1945–54; 1955–64). These partitions consider individuals born in different historical periods which share similar economic and social characteristics. Overall, results from the two different cohort specifications are in line with those from the baseline model. Below, we set out the results from the cohort partition at a 10-year interval.^13^ As shown in Fig. A.3, IOp trends increase with age for each cohort and in most of the countries considered the youngest cohort (1955–64) exhibits higher IOp than older cohorts. This evidence is particularly pronounced in Germany, Sweden, Spain, Italy and Belgium (Fig. 8).

**Fig. 9:**
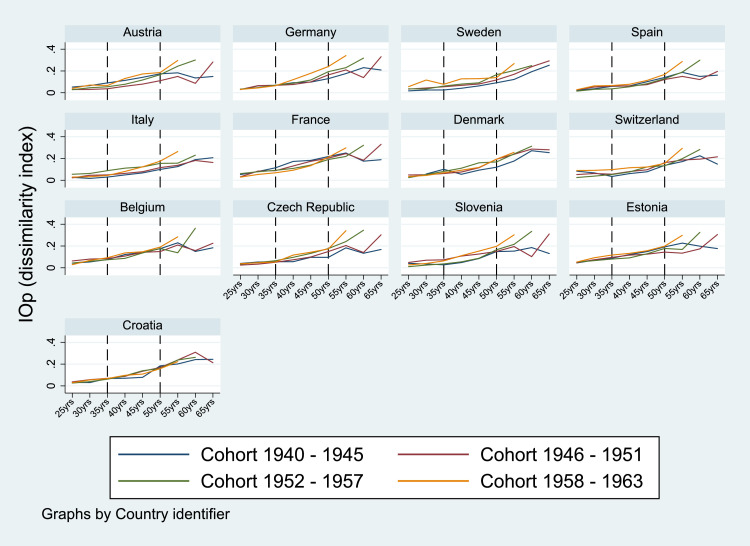
IOp in the prevalence of chronic diseases compared to the country-cohort median, over the life cycle. *Notes:* Authors’ processing of SHARE data, waves 4–7.

Second, in order to test the sensitivity of our results to the choice of the health outcome, we replicate the analysis using an alternative health indicator. Specifically, we create a dummy indicator with value 1 whether the number of chronic conditions reported by individuals is higher than or equal to the median of their cohort at each specific age, and zero otherwise. Compared to the previous indicator (which has value 1 whether respondents report suffering from at least one chronic condition at different life-stages), this variable provides additional information since it compares the health status of a specific individual with the overall health situation of his/her peers, *i.e.*, individuals who are (i) of the same age and (ii) who were born in the same country and cohort. Fig. 9 shows the results. In general, trends in IOp in different countries are very similar to those observed in the baseline specification (Fig. 1): IOp in health increases with age, and is more pronounced in the youngest generation (1955–64).

## 5 Conclusions

This paper investigates the evolution of inequality of opportunity in the incidence of chronic diseases over the lifespan and across different birth cohorts for individuals aged 50 or older in 13 European countries. We adopt an ex-ante parametric approach and rely on the dissimilarity index as our reference inequality metric. In addition to a commonly used set of circumstances, we pay particular attention to the role of adverse childhood events, such as the experience of harm and the quality of the relationship with parents.

Our results provide general evidence of the lasting effects of childhood circumstances on health at different ages, from young adulthood to old age. More in detail, we show that IOp in health is not stable over the life-cycle but generally lower at younger ages and subsequently tending to increase monotonically. Moreover, inequalities seem to be more pronounced for younger cohorts than for older age groups in most of the countries considered.

Regarding circumstances, we find a significant heterogeneity in terms of their contribution to inequality of opportunity in health: ACE account for a significant portion of inequality (from about 25% to 45%), especially at younger ages, while the relative contribution of demographic characteristics (gender) is less pronounced, in particular in mid adulthood (from 30 to 50). Socio-economic conditions such as economic difficulties during childhood and parental education are important sources of IOp, and their contribution remains relatively stable over the lifetime (from about 28% to 45%). It is worth noting that, in general, the impact of early life circumstances related to the parent-child relationship is comparable to that of socio-economic circumstances, while it significantly exceeds the importance of gender, especially in young adulthood. Our findings may have important policy implications. ACE are serious issues since they can importantly contribute to the formation of IOp in health throughout life in the same manner as socio-economic circumstances, with a significant cost not only at individual but also at societal level. In light of this, policy interventions aimed at reducing these inequalities should first identify disadvantaged individuals, who may be considered less responsible for the outcomes observed than better placed individuals, and target them with specific programs such as economic support for families, family-friendly work policies or educational campaigns.

This study has a number of important evidence-based implications, especially in the context of ageing and sustainability. It is widely recognized that inequalities experienced from the earliest years of life and throughout the life course undermine healthy aging. In the context of rapid population ageing, age-related inequalities take on greater urgency. Action on social determinants of health, both across the life course and in early childhood, is needed to reduce inequalities. In addition to policies aimed at influencing health determinants such as health and social care, risk behaviors, and health literacy, interventions in early childhood should be seen as part of a policy makers’ strategy to reduce health inequalities in later life.

Our approach, however, has some intrinsic limitations. First, it does not offer a causal interpretation of the link between adverse circumstances and the prevalence of chronic conditions over the lifespan. At this stage, we aim to understand the relative contribution of a rich set of early life conditions by isolating statistical variations in health attributable to each domain of circumstances, recognizing potential limitations related to (i) the endogeneity of childhood conditions and to (ii) potential underestimation related to unobserved circumstances. Some childhood circumstances are clearly endogenous as there are likely to be unobserved factors that may have influenced both the circumstances and health outcomes of the respondents later in life. More specifically, some childhood conditions, such as adverse health and the absence of a parent, may reflect genetic luck differences, *i.e.*, if parents have a genetic predisposition to some disease, this may influence both the aforementioned circumstances and the health of the children (respondents) later in life. However, even in the presence of unobserved circumstances, our IOp measures can be interpreted as the lower-bound estimates of the overall inequality due to all circumstances, not only those that are observed (see Davillas, Jone, 2020, Ford, Anda, Edwards, Perry, Zhao, Li, Croft, 2011). Second, ACE was retrospectively recalled in adulthood and may have been subject to recall bias and *colouring*. In this regard, Havari and Mazzonna (2015) assessed the internal and external consistency of the measures of childhood health and socio-economic status included in SHARELIFE wave 3 and found that overall, respondents seem to remember their childhood conditions fairly well. Since the method used to collect retrospective information (the Life History Calendar) was also applied in Wave 7, we can plausibly assume that, overall, respondents have a fairly good recollection of their health status and living conditions between ages 0–15. Moreover, some studies note that ACE recollection is relatively accurate (e.g., Hardt, Rutter, 2004, Hardt, Sidor, Bracko, Egle, 2006, Campbell, Conti, Heckman, Moon, Pinto, Pungello, Pan, 2014). Third, since we perform the analysis at a country-cohort level, looking at IOp in specific chronic diseases separately from others may be problematic due to sample size. For instance, the fraction of people who report suffering from mental health rather than arthritis or Alzheimer’s in some countries is very low, especially at younger ages, making it difficult to obtain reliable estimates of IOp. For this reason, we consider an aggregate measure for chronic conditions as an overall proxy for the health status of individuals.

## Declarations

**Funding**: The authors received no financial support for the research, authorship, and publication of this article.

**Conflicts of interest/Competing interests**: None of the authors have actual or potential conflict of interest. The scientific output expressed does not imply a policy position of the European Commission. Neither the European Commission nor any person acting on behalf of the Commission is responsible for the use which might be made of this publication.

**Availability of data and material**: Available upon request.

**Code availability**: Available upon request.

## Figures and Tables

**Fig. A.1:**
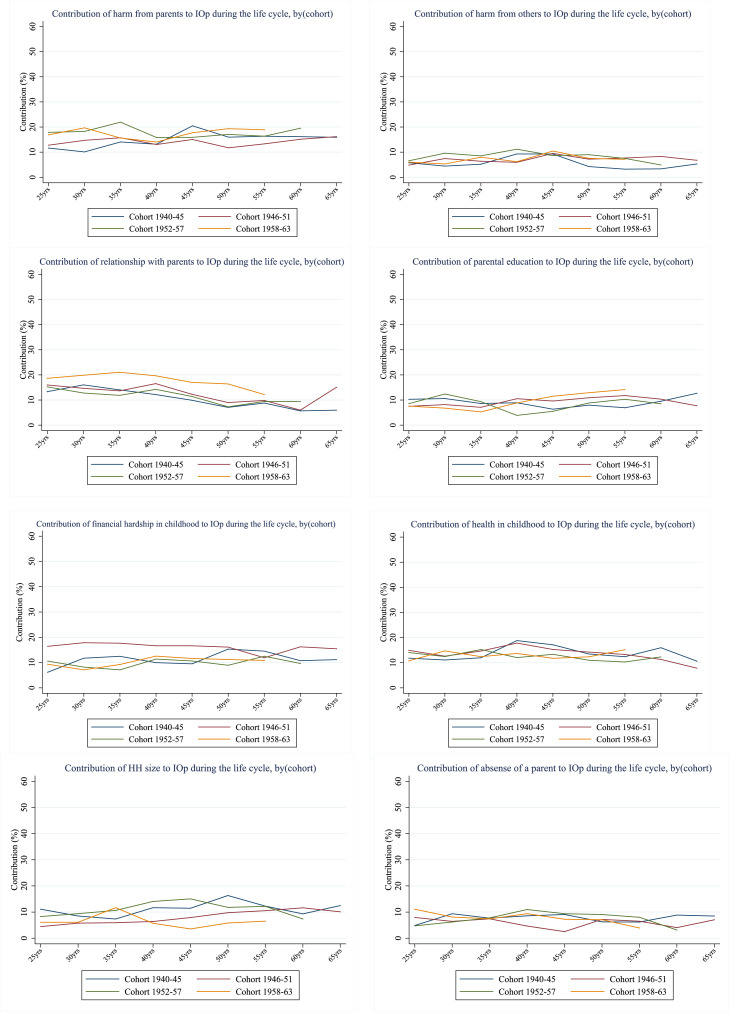
Relative contribution of circumstances (%) over the life-cycle, by cohort (cross-country average). *Notes:* Authors’ processing of SHARE data, waves 4–7.

**Table A.1:** IOp in the prevalence of chronic diseases, by country, cohort and age group.

Country	Life stage (age)	1940–45	1946–51	1952–57	1958–63
Austria	25	.0614803	.035819	.0279615	.0397593
Austria	30	.0708922	.0457407	.0463308	.0732028
Austria	35	.0918894	.0592973	.0625994	.0686885
Austria	40	.1160707	.0717639	.0821391	.1317686
Austria	45	.1448768	.1032128	.1263438	.1809272
Austria	50	.1849913	.1275574	.1735472	.1909532
Austria	55	.2006231	.1778383	.2479091	.3326637
Austria	60	.2251328	.2219474	.3907391	
Austria	65	.2660039	.3350151		
Germany	25	.0300062	.0326195	.0445559	.032104
Germany	30	.0652804	.0670926	.0620964	.0501935
Germany	35	.075401	.0720681	.0814126	.0695016
Germany	40	.107205	.0824393	.1110538	.1219651
Germany	45	.1228252	.112533	.1532892	.1873664
Germany	50	.1706375	.1932314	.2400737	.2625044
Germany	55	.2096425	.2542581	.2853375	.412461
Germany	60	.2668061	.3397786	.4216197	
Germany	65	.3385945	.4552948		
Sweden	25	.0245796	.0340447	.0361126	.055879
Sweden	30	.0309785	.0434683	.0346659	.1194565
Sweden	35	.0318499	.0548574	.061583	.0794089
Sweden	40	.0477284	.0722786	.0801152	.1277206
Sweden	45	.0728796	.0817522	.0963669	.1295563
Sweden	50	.1151998	.1278055	.1774733	.1439963
Sweden	55	.1555292	.1877775	.2135128	.3015913
Sweden	60	.2236428	.272218	.2784075	
Sweden	65	.3020231	.3398685		
Spain	25	.023266	.0318427	.0167256	.0295283
Spain	30	.039036	.0551059	.0352966	.0684762
Spain	35	.0604456	.0640698	.0382323	.0762959
Spain	40	.071734	.0646293	.0602982	.079267
Spain	45	.1021494	.0758715	.0884173	.1179607
Spain	50	.1414962	.1252121	.1469062	.1775316
Spain	55	.1892865	.1639707	.2160558	.3184715
Spain	60	.2297564	.2218828	.3018196	
Spain	65	.2936708	.3095288		
Italy	25	.0277109	.0227887	.0573858	.0293953
Italy	30	.0177586	.0464707	.064255	.0349087
Italy	35	.0295211	.0510421	.089638	.0475966
Italy	40	.0499703	.0753183	.1156267	.0849364
Italy	45	.0676751	.1045864	.1311857	.1338557
Italy	50	.1103632	.14625	.1713721	.1908893
Italy	55	.1451461	.1773782	.1863512	.3086983
Italy	60	.2158229	.2351428	.293417	
Italy	65	.2682876	.3020203		
France	25	.0563776	.032665	.0639138	.0312296
France	30	.0899759	.0846154	.08101	.061761
France	35	.1279031	.088377	.0996127	.0711569
France	40	.1915336	.1316207	.1243424	.0982572
France	45	.2143198	.1771381	.1619972	.1439798
France	50	.2638	.2153989	.2180648	.2252218
France	55	.2975185	.2681907	.2614114	.3907643
France	60	.3046786	.3067903	.410328	
France	65	.3352931	.4341418		
Denmark	25	.4897959	.0499071	.0244641	.034365
Denmark	30	.0657903	.0520815	.0548344	.0446593
Denmark	35	.1068431	.0656656	.083575	.0693388
Denmark	40	.0609014	.0836735	.1138241	.0949921
Denmark	45	.0987157	.1180325	.1602059	.127271
Denmark	50	.1240613	.1973079	.1701895	.2109534
Denmark	55	.184664	.2429627	.2489906	.3110278
Denmark	60	.2720809	.3022081	.3565243	
Denmark	65	.3392461	.3801562		
Switzerland	25	.0853079	.0530975	.0256691	.090252
Switzerland	30	.0682274	.0628389	.0383861	.0920137
Switzerland	35	.0357372	.0569925	.0546232	.0986982
Switzerland	40	.0641384	.0904459	.0766078	.120307
Switzerland	45	.0841028	.1261846	.1242649	.1388781
Switzerland	50	.136852	.1760206	.1417221	.1673335
Switzerland	55	.1769369	.1987337	.2113534	.3073176
Switzerland	60	.2282246	.2202003	.3452505	
Switzerland	65	.2693245	.3586688		
Belgium	25	.0439197	.0629519	.0445419	.0293996
Belgium	30	.0562984	.0805043	.0609225	.0718225
Belgium	35	.0764239	.0855586	.0745042	.0967526
Belgium	40	.1229588	.1097834	.0982376	.1476284
Belgium	45	.1408122	.149845	.1420581	.1624833
Belgium	50	.1784688	.1657111	.1886083	.220912
Belgium	55	.2327964	.2231876	.2467517	.3577627
Belgium	60	.2388602	.2749009	.3778084	
Belgium	65	.2716444	.3308335		
Czech Republic	25	.040456	.0267197	.0346475	.0383482
Czech Republic	30	.0561398	.0367359	.0492144	.0462804
Czech Republic	35	.0690027	.0516463	.0652579	.0536776
Czech Republic	40	.0810774	.0763787	.0997435	.118398
Czech Republic	45	.1185207	.1059439	.1339351	.1466027
Czech Republic	50	.1352987	.1686349	.1866791	.2044112
Czech Republic	55	.2022296	.2329953	.2521623	.3902428
Czech Republic	60	.2640058	.2959386	.3875879	
Czech Republic	65	.3025705	.4087274		
Slovenia	25	.0418885	.0502741	.0109843	.0278599
Slovenia	30	.0375763	.0700576	.0328191	.039942
Slovenia	35	.0321465	.0733918	.0542182	.0641983
Slovenia	40	.0554329	.1095697	.067176	.1129111
Slovenia	45	.0947656	.1265776	.0973105	.1552235
Slovenia	50	.158956	.152579	.1786693	.2162947
Slovenia	55	.1776513	.2033389	.2389041	.375991
Slovenia	60	.2413909	.2618763	.4000743	
Slovenia	65	.2804586	.3946575		
Estonia	25	.0458201	.0508474	.0522948	.055831
Estonia	30	.071757	.0749357	.0662406	.0979355
Estonia	35	.0911433	.0969171	.0815938	.1220112
Estonia	40	.1243734	.1166101	.0994357	.1406821
Estonia	45	.1504194	.1275851	.1378538	.1880966
Estonia	50	.194434	.1734401	.1959654	.2652466
Estonia	55	.2291003	.2304365	.2805864	.4120203
Estonia	60	.2717029	.2825159	.4204348	
Estonia	65	.3103417	.4303541		
Croatia	25	.037413	.0368683	.0265388	.0307786
Croatia	30	.034333	.0571612	.04027	.0567498
Croatia	35	.078405	.0693117	.0629928	.0733164
Croatia	40	.0862577	.0908647	.0969709	.1107978
Croatia	45	.1133395	.1443275	.1493549	.1428021
Croatia	50	.2070621	.1974129	.1983126	.22708
Croatia	55	.252012	.2706959	.2733095	.3114606
Croatia	60	.3281065	.3512276	.3360557	
Croatia	65	.3779794	.3917651		

*Notes:* Authors’ processing of SHARE data, waves 4–7.

**Table A.2:** Summary statistics.

Variable	Mean	Std. Dev.	Min.	Max.	N
chronic25	0.029	0.168	0	1	117884
chronic30	0.053	0.224	0	1	117884
chronic35	0.074	0.262	0	1	117884
chronic40	0.121	0.327	0	1	117884
chronic45	0.177	0.381	0	1	117884
chronic50	0.28	0.449	0	1	117884
chronic55	0.369	0.482	0	1	106964
chronic60	0.455	0.498	0	1	85280
chronic65	0.533	0.499	0	1	61947
Age	66.869	9.636	50	100	117884
Female	0.570	0.495	0	1	117884
HH size when 10yrs	5.236	1.969	1	15	117884
Poor family when 10yrs	0.242	0.428	0	1	117884
Parents tertiary	0.137	0.343	0	1	117884
Absent parent when 10yrs	0.139	0.346	0	1	117884
Adverse health when 10yrs	0.123	0.328	0	1	117884
Harm (third parties)	0.06	0.238	0	1	117884
Harm (parents)	0.246	0.431	0	1	117884
Adverse relationship (parents)	0.206	0.405	0	1	117884

*Notes*: Authors’ processing of SHARE data, waves 4–7. “Chronic25 - chronic65” indicate dummy variables that equal 1 if an individual reports suffering from at least one chronic disease at different ages. Missing observations for chronic diseases at the ages of 55, 60, and 65 refer to individuals younger than 55, 60 or 65 at the moment of the interview.

**Table A.3:** The prevalence of single health problems by country.

40 yrs	heart	stroke	diab	lung	cancer	park	alzh	emot	kidney
Austria	0.0082	0.0026	0.0093	0.0047	0.0064	0.0000	0.0000	0.0000	0.0000
Germany	0.0106	0.0021	0.0131	0.0177	0.0113	0.0004	0.0004	0.0223	0.0000
Sweden	0.0050	0.0016	0.0089	0.0066	0.0071	0.0000	0.0000	0.0048	0.0000
Spain	0.0054	0.0007	0.0130	0.0135	0.0051	0.0000	0.0004	0.0078	0.0000
Italy	0.0064	0.0012	0.0122	0.0093	0.0032	0.0000	0.0000	0.0082	0.0011
France	0.0095	0.0016	0.0076	0.0071	0.0050	0.0006	0.0006	0.0044	0.0003
Denmark	0.0062	0.0015	0.0070	0.0084	0.0135	0.0004	0.0000	0.0077	0.0000
Switzerland	0.0075	0.0022	0.0064	0.0044	0.0057	0.0000	0.0000	0.0017	0.0000
Belgium	0.0113	0.0037	0.0101	0.0121	0.0076	0.0000	0.0001	0.0146	0.0007
Czech R.	0.0131	0.0031	0.0120	0.0110	0.0048	0.0003	0.0000	0.0000	0.0001
Slovenia	0.0132	0.0031	0.0122	0.0082	0.0041	0.0000	0.0003	0.0046	0.0018
Estonia	0.0368	0.0032	0.0049	0.0205	0.0045	0.0012	0.0002	0.0014	0.0006
Croatia	0.0175	0.0016	0.0083	0.0083	0.0036	0.0000	0.0010	0.0161	0.0062
Total	0.0130	0.0023	0.0097	0.0111	0.0061	0.0003	0.0002	0.0066	0.0007
	hyper	chol	asthma	arth	osteo	ulcer	psych	cardio	chronic40
Austria	0.0587	0.0438	0.0077	0.0285	0.0017	0.0202	0.0000	0.0107	0.1314
Germany	0.0669	0.0327	0.0000	0.0005	0.0000	0.0185	0.0222	0.0124	0.1257
Sweden	0.0371	0.0115	0.0048	0.0025	0.0000	0.0050	0.0048	0.0065	0.0682
Spain	0.0297	0.0203	0.0000	0.0196	0.0000	0.0180	0.0078	0.0061	0.1013
Italy	0.0389	0.0204	0.0004	0.0216	0.0010	0.0164	0.0082	0.0076	0.0968
France	0.0652	0.0400	0.0011	0.0600	0.0000	0.0154	0.0044	0.0104	0.1503
Denmark	0.0415	0.0129	0.0008	0.0122	0.0000	0.0166	0.0077	0.0077	0.0948
Switzerland	0.0359	0.0164	0.0000	0.0304	0.0000	0.0101	0.0017	0.0097	0.0853
Belgium	0.0621	0.0395	0.0052	0.0388	0.0027	0.0315	0.0145	0.0143	0.1672
Czech R.	0.0561	0.0163	0.0000	0.0201	0.0000	0.0245	0.0000	0.0153	0.1081
Slovenia	0.0585	0.0197	0.0000	0.0123	0.0000	0.0249	0.0046	0.0160	0.1077
Estonia	0.0785	0.0122	0.0000	0.0480	0.0000	0.0344	0.0014	0.0395	0.1701
Croatia	0.0637	0.0167	0.0000	0.0000	0.0000	0.0198	0.0161	0.0184	0.1020
Total	0.0552	0.0236	0.0015	0.0248	0.0005	0.0214	0.0066	0.0150	0.1213
60 yrs	heart	stroke	diab	lung	cancer	park	alzh	emot	kidney
Austria	0.0673	0.0291	0.0886	0.0410	0.0405	0.0015	0.0047	0.0110	0.0005
Germany	0.0759	0.0285	0.0989	0.0614	0.0655	0.0040	0.0045	0.0927	0.0005
Sweden	0.0494	0.0226	0.0547	0.0292	0.0472	0.0021	0.0018	0.0173	0.0002
Spain	0.0555	0.0121	0.0992	0.0417	0.0395	0.0020	0.0024	0.0496	0.0018
Italy	0.0543	0.0141	0.0788	0.0385	0.0354	0.0004	0.0011	0.0315	0.0033
France	0.0734	0.0217	0.0815	0.0463	0.0403	0.0020	0.0025	0.0244	0.0013
Denmark	0.0553	0.0301	0.0456	0.0460	0.0544	0.0015	0.0000	0.0437	0.0006
Switzerland	0.0467	0.0099	0.0407	0.0284	0.0385	0.0011	0.0031	0.0112	0.0004
Belgium	0.0677	0.0247	0.0817	0.0562	0.0510	0.0010	0.0045	0.0498	0.0034
Czech R.	0.0976	0.0286	0.1203	0.0544	0.0395	0.0034	0.0035	0.0140	0.0008
Slovenia	0.0805	0.0236	0.0888	0.0369	0.0375	0.0007	0.0067	0.0254	0.0050
Estonia	0.1461	0.0269	0.0786	0.0604	0.0412	0.0032	0.0032	0.0138	0.0021
Croatia	0.0907	0.0302	0.0952	0.0320	0.0411	0.0005	0.0049	0.0548	0.0198
Total	0.0789	0.0234	0.0838	0.0462	0.0437	0.0020	0.0034	0.0326	0.0025
	hyper	chol	asthm	arth	osteo	ulcer	psych	cardio	chronic60
Austria	0.3375	0.2203	0.0202	0.1104	0.0341	0.0454	0.0034	0.0871	0.5056
Germany	0.3545	0.1905	0.0000	0.0037	0.0010	0.0375	0.0616	0.1057	0.4298
Sweden	0.2614	0.1210	0.0096	0.0124	0.0021	0.0190	0.0116	0.0708	0.3310
Spain	0.2663	0.2324	0.0000	0.0877	0.0024	0.0444	0.0340	0.0639	0.4415
Italy	0.3148	0.1993	0.0035	0.0863	0.0054	0.0491	0.0226	0.0729	0.4564
France	0.2799	0.2227	0.0044	0.1951	0.0008	0.0358	0.0061	0.0897	0.4879
Denmark	0.2430	0.1912	0.0021	0.0485	0.0006	0.0371	0.0343	0.0765	0.3534
Switzerland	0.2169	0.1338	0.0000	0.1247	0.0000	0.0253	0.0049	0.0552	0.3364
Belgium	0.2939	0.2868	0.0122	0.1330	0.0229	0.0828	0.0324	0.0836	0.5139
Czech R.	0.4103	0.2024	0.0000	0.0964	0.0000	0.0549	0.0066	0.1195	0.5041
Slovenia	0.3589	0.2155	0.0000	0.0439	0.0000	0.0681	0.0191	0.0992	0.4478
Estonia	0.3758	0.1577	0.0000	0.1895	0.0000	0.0938	0.0035	0.1762	0.5213
Croatia	0.3697	0.1677	0.0000	0.0000	0.0000	0.0541	0.0393	0.1030	0.4236
Total	0.3224	0.1994	0.0040	0.0961	0.0059	0.0543	0.0192	0.0998	0.4555

*Notes:* Authors’ processing of SHARE data, waves 4–7. All the variables are binary. The list of chronic problems includes: heart diseases, including angina, heart attack or other heart problems (“hearth”); stroke; diabetes or high blood sugar (“diab”); lung disease other than asthma such as bronchitis; chronic obstructive pulmonary disease or tuberculosis (“lung”); cancer or malignant tumour (“cancer”); Parkinson’s disease (“park”); Alzheimer’s disease (“alzh”); emotional distress (“emot”); kidney disease (“kidney”); high blood pressure (“hyper”); high cholesterol (“chol”); asthma; arthritis, including osteoarthritis and rheumatism (“arth”); osteoporosis (“osteo”); ulcer; nervous, or psychiatric problem (“psych”); cardio-vascular diseases (“cardio”). The variables “chronic40” and “chronic60” are dummy indicators, assigning value 1 whether an individual reports suffering from at least one chronic condition at the age of 40 or 60 respectively.

## Footnotes

1 For more details see: https://www.euro.who.int/_data/assets/pdf_file/0006/199536/Health2020-Short.pdf.

2 Although SAH has proved effective in predicting mortality and health-care utilisation (van Doorslaer and Gerdtham, 2003), it has some limitations. An important one is reporting bias: sub-groups of the population may in fact use different thresholds when assessing their health status, even if their objective health conditions are likely the same.

3 We are aware that chronic diseases may occur at different life-stages (such as arthritis and Alzheimer’s disease at older ages) and that the mechanisms relating early life conditions to health might differ across specific health categories (such as mental and physical health problems). However, we are not able to look at IOp for specific classes of illnesses because the incidence of several chronic problems is skewed towards zero, especially at younger ages. See Table A.3 in the Appendix.

4 Results do not vary substantially when the other dummy variable indicating a situation of “child neglect” is included in the model.

5 In the case of a 10-year cohort partition, the number of individuals increases to 106,282.

6 The interpretation of the results related to the youngest cohort in Sweden require some caution since the cohort balance requirement is only partially satisfied.

7 This method is known as the *ex-ante* approach to IOp. It is based on the principle that there is equality of opportunity if all individuals face the same opportunity set, prior to their efforts and outcomes being realised (Fleurbaey and Schokkaert, 2009). The ex-ante approach suggests that all individuals have equal opportunities if no differences in outcomes emerge from having different circumstances.

8 It is important to note that this effect is not due to scaling, *i.e.,* the fact that absolute measures of inequality increase with a higher prevalence of chronic conditions at older ages. IOp captures the extent of the inequality of circumstances’ contributions, which does not necessarily become more unequal when the overall prevalence of chronic conditions increases.

9 For more details see: https://www.statista.com/statistics/730758/western-europe-economic-manufacturing-output-growth-golden-age/.

10 For more details, see https://doi.org/10.1787/9789264300040-en.

11 In this section we report the average contribution of ACE (harm from parents, harm from others, and relationship with parents) and socio-economic circumstances (parental education, financial hardship, health in childhood, household size, and absence of a parent) at the aggregate level. In the appendix we report the average contributions of each single circumstance separately.

12 There is an extensive literature which shows a persistent relationship between the socio-economic status of parents and the socio-economic outcomes of their children as adults (for a detailed review, see Blanden, 2013).

13 Results from partitions at 5 and 6-year intervals are available from the authors on request.
